# Hyponatraemia-induced Takotsubo syndrome secondary to idiopathic syndrome of inappropriate antidiuretic hormone: a case report

**DOI:** 10.1093/ehjcr/ytaf006

**Published:** 2025-01-10

**Authors:** Felipe Israel López-Trejo, Martin Rodrigo Cedillo-Urbina, Andrea Paulina Maldonado-Tenesaca, Juan Carlos Rivera-Guerrero, Elias Noel Andrade-Cuellar

**Affiliations:** Department of Cardiology, National Medical Center ‘November 20th’, ISSSTE, Av. Felix Cuevas #540, Col. Del Valle Del. Benito Juarez, C.P. 03100 Mexico City, Mexico; Department of Cardiology, National Medical Center ‘November 20th’, ISSSTE, Av. Felix Cuevas #540, Col. Del Valle Del. Benito Juarez, C.P. 03100 Mexico City, Mexico; Echocardiography Laboratory, National Medical Center ‘November 20th’, ISSSTE, Av. Felix Cuevas #540, Col. Del Valle Del. Benito Juarez, C.P. 03100 Mexico City, Mexico; Department of Cardiology, National Medical Center ‘November 20th’, ISSSTE, Av. Felix Cuevas #540, Col. Del Valle Del. Benito Juarez, C.P. 03100 Mexico City, Mexico; Department of Cardiology, National Medical Center ‘November 20th’, ISSSTE, Av. Felix Cuevas #540, Col. Del Valle Del. Benito Juarez, C.P. 03100 Mexico City, Mexico; Universidad Nacional Autónoma de México, Escolar 411A, Copilco Universidad, Coyoacán, C.P. 04360 Mexico City, Mexico; Cardiac Electrophysiology, National Medical Center ‘November 20th’, ISSSTE, Av. Felix Cuevas #540, Col. Del Valle Del. Benito Juarez, C.P. 03100 Mexico City, Mexico

**Keywords:** Takotsubo cardiomyopathy, Hyponatraemia, Stress cardiomyopathy, SIADH, Left ventricular dysfunction, Case report

## Abstract

**Background:**

Takotsubo cardiomyopathy (TC), also known as stress cardiomyopathy or broken-heart syndrome, is an acute, reversible left ventricular dysfunction often triggered by stress and associated with elevated catecholamine levels. Hyponatraemia has been recognized as a potential trigger for TC, although its pathophysiological mechanisms remain unclear.

**Case summary:**

We present the case of an 84-year-old woman with a history of Type 2 diabetes, primary hypertension, hypothyroidism, and osteoporosis, who was admitted for typical angina and seizures. Initial laboratory tests revealed severe hyponatraemia (serum sodium: 119 mmol/L) and elevated cardiac enzymes. Electrocardiogram showed Wellens Type B syndrome and subsequent ST-segment elevation. Coronary angiography revealed no significant coronary lesions. Optical coherence tomography showed a non-significant lesion in the anterior descending artery, and ventriculography confirmed TC. The patient was treated with beta-blockers and managed for syndrome of inappropriate antidiuretic hormone-induced hyponatraemia with fluid restriction. Follow-up echocardiography at three months showed recovery of global longitudinal strain and preserved biventricular systolic function. Cardiac magnetic resonance imaging demonstrated normal chamber dimensions and late gadolinium enhancement with a non-ischaemic pattern.

**Discussion:**

This case underscores the association between severe hyponatraemia and TC, highlighting the need for careful electrolyte management in patients with acute cardiac symptoms. The exact mechanisms linking hyponatraemia and TC remain to be elucidated, but this case contributes to the limited literature on hyponatraemia-induced TC, emphasizing the importance of recognizing and addressing this electrolyte imbalance to prevent cardiac complications.

**Learning points**

- Severe hyponatraemia, particularly in the context of syndrome of inappropriate antidiuretic hormone, can act as a trigger for Takotsubo cardiomyopathy, highlighting the critical importance of monitoring and addressing electrolyte imbalances in patients presenting with acute cardiac symptoms.
- This case underscores the necessity of further research into the pathophysiology of hyponatraemia-induced Takotsubo cardiomyopathy, with particular attention to the roles of catecholamines and myocardial stunning mechanisms, to optimize clinical management and improve patient outcomes.
- The integration of clinical context, laboratory findings, electrocardiogram, and both invasive and non-invasive imaging modalities is essential for the accurate diagnosis of Takotsubo cardiomyopathy.

## Introduction

Takotsubo cardiomyopathy (TC), also known as stress-induced cardiomyopathy, is characterized by transient systolic dysfunction of the left ventricle, often triggered by emotional or physical stress. While it mimics acute coronary syndrome (ACS), it typically presents without significant coronary artery obstruction. The condition is predominantly seen in postmenopausal women and is frequently associated with elevated catecholamine levels, which are believed to contribute to myocardial stunning and transient left ventricular dysfunction.^1^

Severe hyponatraemia has been identified as a rare potential trigger for TC. Hyponatraemia may exacerbate the vulnerability of the myocardium by altering cellular excitability and intracellular calcium handling, thus increasing susceptibility to stress-induced myocardial injury.^2^ In some cases, hyponatraemia is linked to the syndrome of inappropriate antidiuretic hormone (SIADH) secretion, which leads to hypoosmolar hyponatraemia due to excessive ADH release. This report presents a case of hyponatraemia-induced TC secondary to idiopathic SIADH in an elderly woman.

## Summary figure

**Figure:**
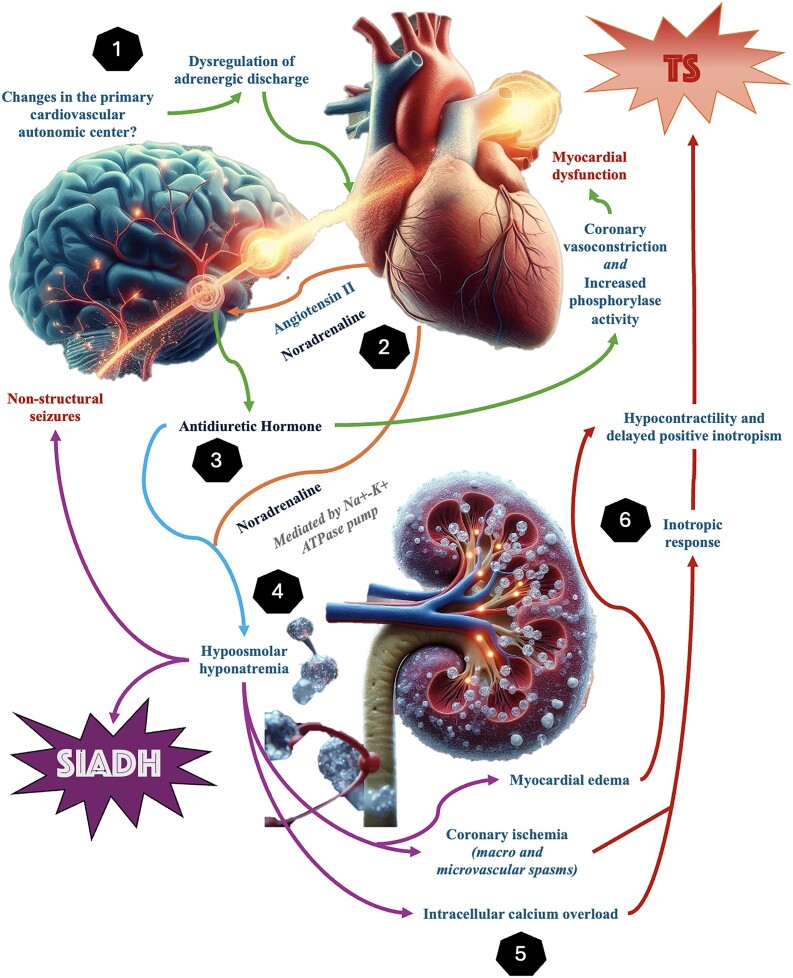


## Case presentation

Our patient is an 84-year-old overweight woman with a medical history of Type 2 diabetes, primary arterial hypertension, hypothyroidism, and osteoporosis. She was being treated with levothyroxine, metformin, linagliptin, losartan, indapamide, pregabalin, calcitriol, and denosumab. One month before admission, she had normal sodium levels documented during a routine endocrinology follow-up. She presented to the emergency department with anginal chest pain and two episodes of epileptic seizures.

On initial assessment, her vital signs were stable, with a blood pressure of 110/70 mmHg and a heart rate of 85 beats per minute. Cardiovascular examination revealed normal jugular venous pressure, no peripheral oedema, and normal heart sounds without murmurs or gallops, indicating a euvolaemic status. Initial laboratory tests revealed a serum sodium level of 119 mmol/L (normal range: 135–145 mmol/L), NT-proBNP: 53 175 pg/mL (normal < 125 pg/mL), and TnI-Hs: 1657 ng/L (normal < 14 ng/L). The complete blood count, liver and renal function, thyroid function, and cortisol levels were within normal ranges. The initial electrocardiogram (ECG) was unremarkable, showing sinus rhythm without ST-segment or T-wave abnormalities (see Supplementary material online, *Figure S1*). A cranial CT scan ruled out structural brain abnormalities (see Supplementary material online, *Video S1*), and the patient was treated with 3% hypertonic saline solution (60 mL of 20% saline in 500 mL of 0.9% saline), at an infusion rate of 1 mL/kg/h to correct the severe hyponatraemia. The sodium level was corrected gradually, targeting an increase to >130 mmol/L over 48 h, with careful monitoring to avoid rapid overcorrection.

**Figure 1:**
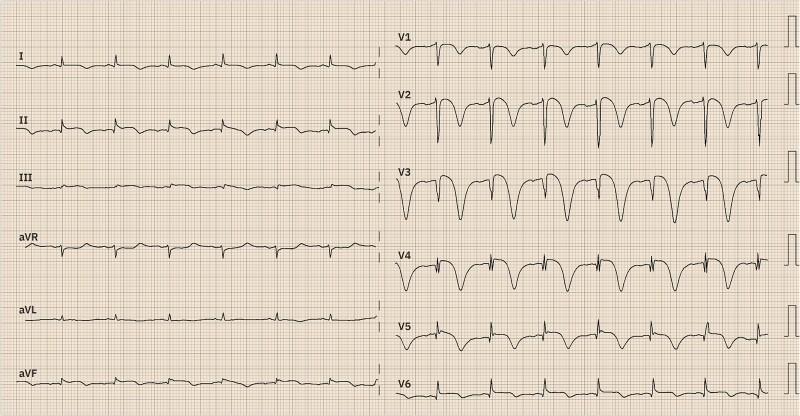
Twelve-lead ECG showing anterolateral and inferior ST-segment elevations, T wave inversion, and QTc prolongation consistent with Wellens Type B pattern.

**Figure 2:**
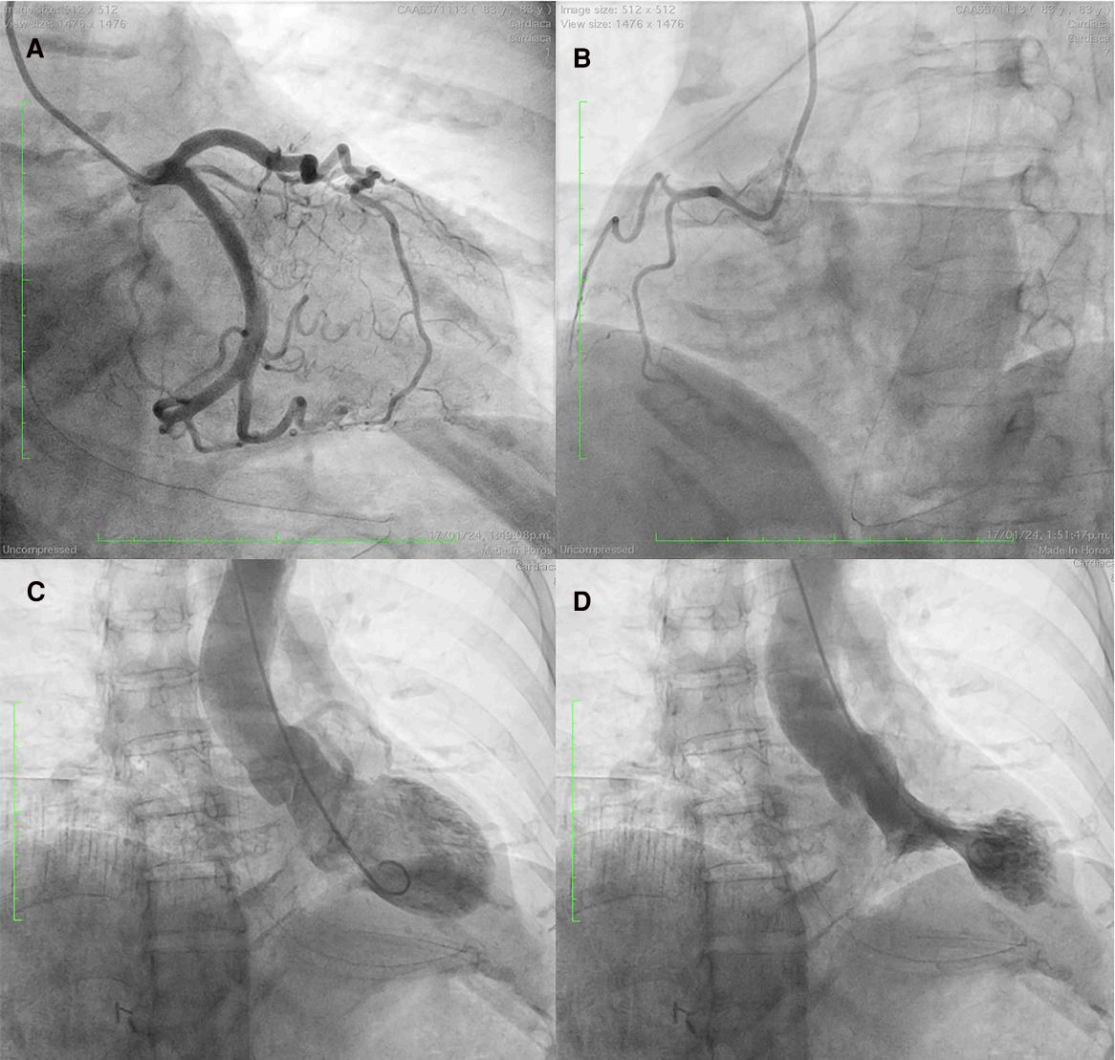
Coronary angiography (*A*) showing no significant stenosis and ventriculography (*B*) demonstrating apical ballooning with mid and basal hyperkinesia.

Approximately 48 h after admission, an ECG revealed Wellens Type B syndrome, characterized by anatomically heterogeneous ST–T wave changes, including ST-segment elevations in the anterolateral and inferior leads. These findings make the involvement of a specific coronary artery territory less likely (*Figure 1*). The patient was initially hospitalized in a regular cardiology ward and was transferred to the coronary care unit upon the detection of ECG changes. Urgent coronary angiography showed no significant coronary artery stenosis (*Figure 2A* and *B*, Supplementary material online, *Videos S2* and *S3*). Given the ECG findings, optical coherence tomography was performed with a Dragonfly® catheter, showing a non-significant lesion in the LAD. Left ventriculography demonstrated apical ballooning with mid and basal hyperkinesia, confirming the diagnosis of TC (*Figure 2C* and *D* and Supplementary material online, *Video S4*). The patient remained haemodynamically stable throughout the procedure with no signs of shock or heart failure.

**Figure 3:**
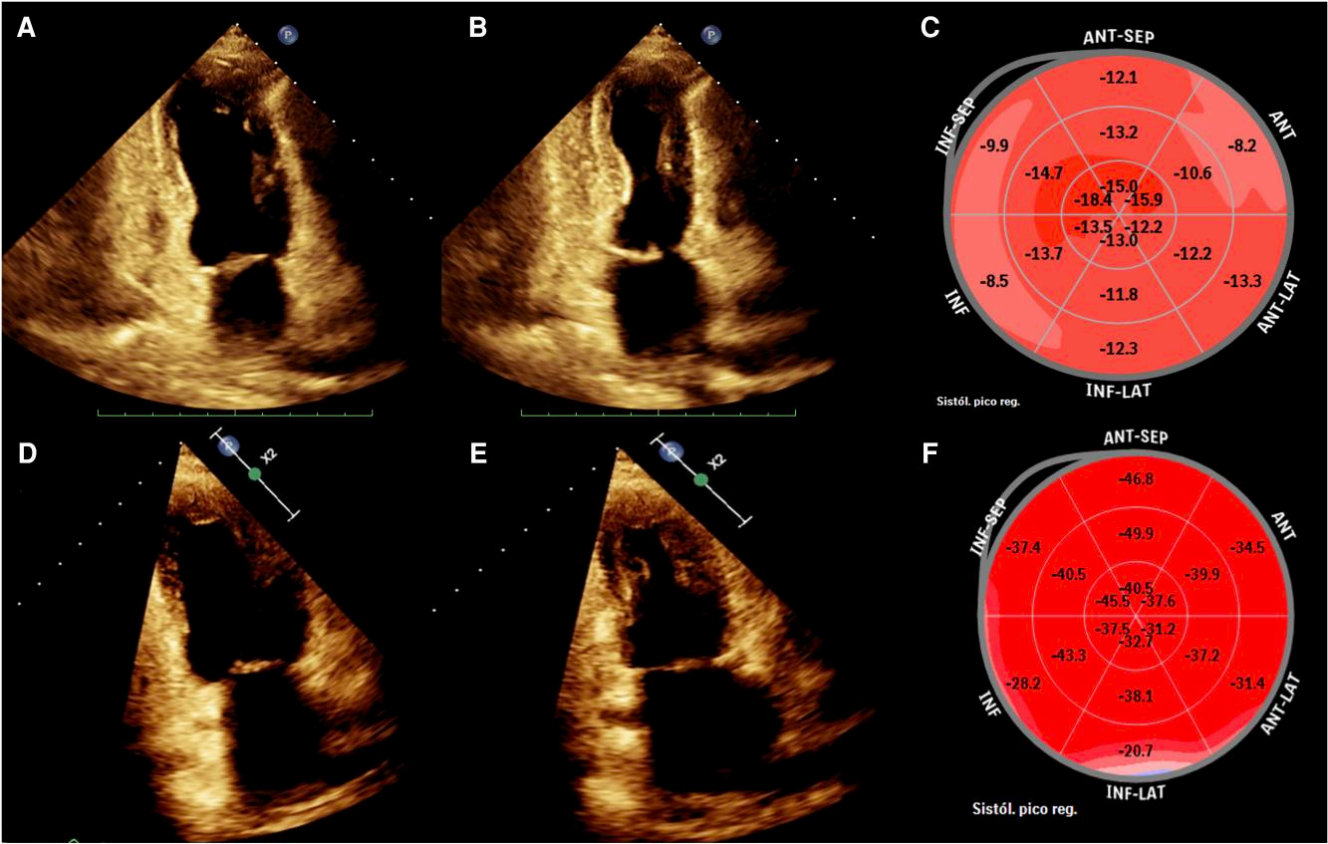
Transthoracic echocardiogram during the acute event (*A* and *B*) showing basal hyperkinesia and apical akinesia, and at the three-month follow-up (*D–F*) demonstrating normalization of regional wall motion. Note that the GLS was reduced in the basal segments, which were otherwise normal, compared to the apical segment where there was akinesia during the acute event (*C*). This may be secondary to disproportionate exposure or an exaggerated response to catecholamines in the basal segments, leading to temporary dysfunction or reduced contractility, as reflected by the reduced strain in these segments.

The initial echocardiography revealed an ejection fraction of 40%, hyperkinesia of the basal segments with apical akinesia, and a global longitudinal strain (GLS) of −12%. No valvular heart disease or left ventricular outflow tract obstruction was noted (*Figure 3A–C*, Supplementary material online, *Video S5*). The diagnosis of idiopathic SIADH was established based on a urine osmolarity of 720 mOsm/kg and a urine sodium concentration of 65 mmol/L, with exclusion of other causes such as diuretic use, hypothyroidism, or renal dysfunction. To rule out a potential paraneoplastic cause of the hyponatraemia, a non-contrast chest CT scan (see Supplementary material online, *Video S6*) was performed, which revealed no findings suggestive of underlying neoplasms.

**Figure 4:**
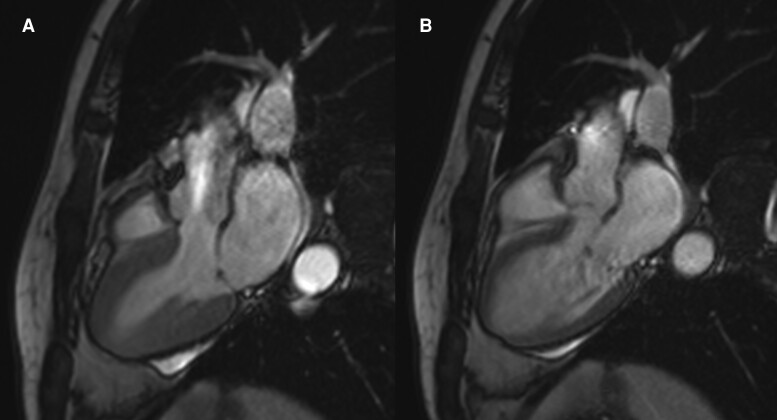
Cardiac MRI at three-month follow-up. A short-axis view is shown in end-systole (*A*) and end-diastole (*B*), revealing the absence of morphological abnormalities and apical dyskinesia.

The patient was started on carvedilol 6.25 mg twice daily and continued on fluid restriction. Over the following days, her sodium levels normalized, and her clinical condition improved significantly. She was discharged with carvedilol and continued fluid restriction. At three months, repeat echocardiography showed complete resolution of the apical akinesia and normalization of GLS (*Figure 3D–F*; Supplementary material online, *Video S7*), and cardiac magnetic resonance imaging (MRI) demonstrated the absence of morphological abnormalities and apical dyskinesia (*Figure 4* and Supplementary material online, *Video S8*), along with late gadolinium enhancement (LGE) in the intramyocardial septum, suggesting incomplete reversibility (see Supplementary material online, *Video S9*).

## Discussion

To our knowledge, there are few case reports in the literature on hyponatraemia-induced TC. Our case adds to this limited body of evidence, demonstrating a clear association between severe hyponatraemia secondary to SIADH and the development of TC.

In elderly patients with multiple comorbidities, hyponatraemia is often multifactorial, frequently related to diuretics, hypothyroidism, or kidney disease. In this case, these were excluded, confirming idiopathic SIADH. Previous reports have documented similar scenarios where severe hyponatraemia, often secondary to SIADH, has precipitated TC (*Table 1*). One study described a 76-year-old woman with severe hyponatraemia due to diuretic therapy who presented with confusion and agitation. Her ECG showed ST-segment elevation, and subsequent imaging confirmed TC, which resolved after correcting the hyponatraemia.^3^ Another case involved a 57-year-old woman where TC was triggered by hyponatraemia secondary to SIADH, and her condition improved significantly following fluid restriction and electrolyte correction.^2^

**Table 1:** Comparative table of existing case reports in the literature with Takotsubo syndrome and SIADH

Features of published cases with hyponatraemia-induced Takotsubo syndrome (TTS) secondary to idiopathic syndrome of inappropriate antidiuretic hormone (SIADH)									
Reference	Age	Gender	Serum sodium level	ECG findings	TS morphology	LV function	Neurological findings	Coronary angiography findings	Definition
Murguía-Aranda A, *et al.*	85 years	Female	117 mEq/L	LBBB and ST-segment elevation in V1–V3	Apical ballooning	Apical and midventricular akinesis LVEF: 30%	Aphasia	60% stenosis in the LAD artery, 40% in-stent stenosis in the OM branch	TS with by-stander hyponatraemia
Jha KK, *et al.*	55 years	Female	108 mEq/L	Precordial T wave inversion	Apical ballooning	Akinesis of the apical myocardium LVEF: 20%–25%	Frontal headache	Non-obstructive	Hyponatraemia-induced TS
AbouEzzeddine O, *et al.*	57 years	Female	111 mEq/L	Non-abnormality	Apical ballooning	Apical and midventricular akinesis LVEF: 35%	None	Non-obstructive	Hyponatraemia-induced TS
Lopez-Trejo FI, *et al.*	84 years	Female	124 mEq/L	Type B Wellens’s syndrome and consecutively anterolateral ST-segment elevation with QTc prolongation	Apical ballooning	Medial and basal hyperkinesia with apical akinesia LVEF: 50% SGL: −16.1%	Seizures	Non-obstructive	Hyponatraemia-induced TS

The pathophysiological mechanisms linking hyponatraemia to TC are complex. Elevated catecholamine levels, a hallmark of TC, can lead to direct myocardial injury or induce coronary vasospasm, contributing to transient left ventricular dysfunction.^1,4^ Hyponatraemia may exacerbate myocardial vulnerability by impairing intracellular calcium homeostasis, increasing cellular excitability, and intensifying adrenergic stimulation. Additionally, osmotic triggers and adrenergic substances such as noradrenaline may induce the release of ADH from the supraoptic nucleus, leading to hypoosmolar hyponatraemia and myocardial dysfunction through vasoconstriction and phosphorylase activity, resulting in glycogen depletion.^5^

The differential diagnosis of TC includes ACS and acute myocarditis, both of which can present with chest pain and ST-segment elevation. Acute coronary syndrome typically involves localized ischaemia from coronary artery obstruction, which was ruled out in our patient through coronary angiography (*Figure 2A* and *B*). Acute myocarditis can mimic TC, with viral infections often implicated, but the absence of myocardial oedema and the rapid improvement in function helped exclude this diagnosis.^6^ Hypertrophic cardiomyopathy, while presenting with apical dysfunction, generally shows long-standing ECG changes and morphological hypertrophy, which were absent in this case.

Our case underscores the importance of recognizing SIADH as a potential trigger for TC, particularly in patients with significant comorbidities and continuous stressors. Severe hyponatraemia can induce seizures through its effects on neuronal excitability, which may contribute to the development of TC by increasing adrenergic tone.^7^ The presence of seizures in our patient likely exacerbated the stress response, further contributing to TC.

At the three-month follow-up, the cardiac MRI of our patient showed preserved biventricular systolic function, no motion abnormalities, and LGE with a non-ischaemic intramyocardial septal pattern. This suggests incomplete reversibility of myocardial injury, aligning with previous reports documenting subclinical changes even after functional recovery.^6^

In conclusion, there is a possible causal association between hyponatraemia and TC due to mechanisms that have not yet been fully elucidated. Clinicians should be alert to the potential diagnosis and prevention through strict hydro-electrolyte control. Future studies should focus on further elucidating the pathophysiological mechanisms and exploring therapeutic strategies to mitigate the risk of TC in patients with hyponatraemia.

## Supplementary Material

ytaf006_Supplementary_Data

## Data Availability

The data underlying this article are available in the article and in its online Supplementary material.

## Lead author biography

Dr Felipe Israel López-Trejo is a physician graduated from the Escuela Superior de Medicina at the Instituto Politécnico Nacional. He completed his specialty in Internal Medicine at the Faculty of Medicine of the Universidad Nacional Autónoma de México and is currently a Cardiology resident at the same institution. Areas of interest include heart failure, cardiotoxicity, and structural heart disease.

## Notes

**Consent:** Informed consent was obtained from the patient in accordance with COPE guidelines.

**Funding:** Universidad Nacional Autónoma de México.
