# Supplementary figures and images for: Hyponatraemia-induced Takotsubo syndrome secondary to idiopathic syndrome of inappropriate antidiuretic hormone: a case report
Source: Eur Heart J Case Rep. 2025 Jan 10;9(2):ytaf006. doi: 10.1093/ehjcr/ytaf006 (PMC11804244; doi:10.1093/ehjcr/ytaf006)

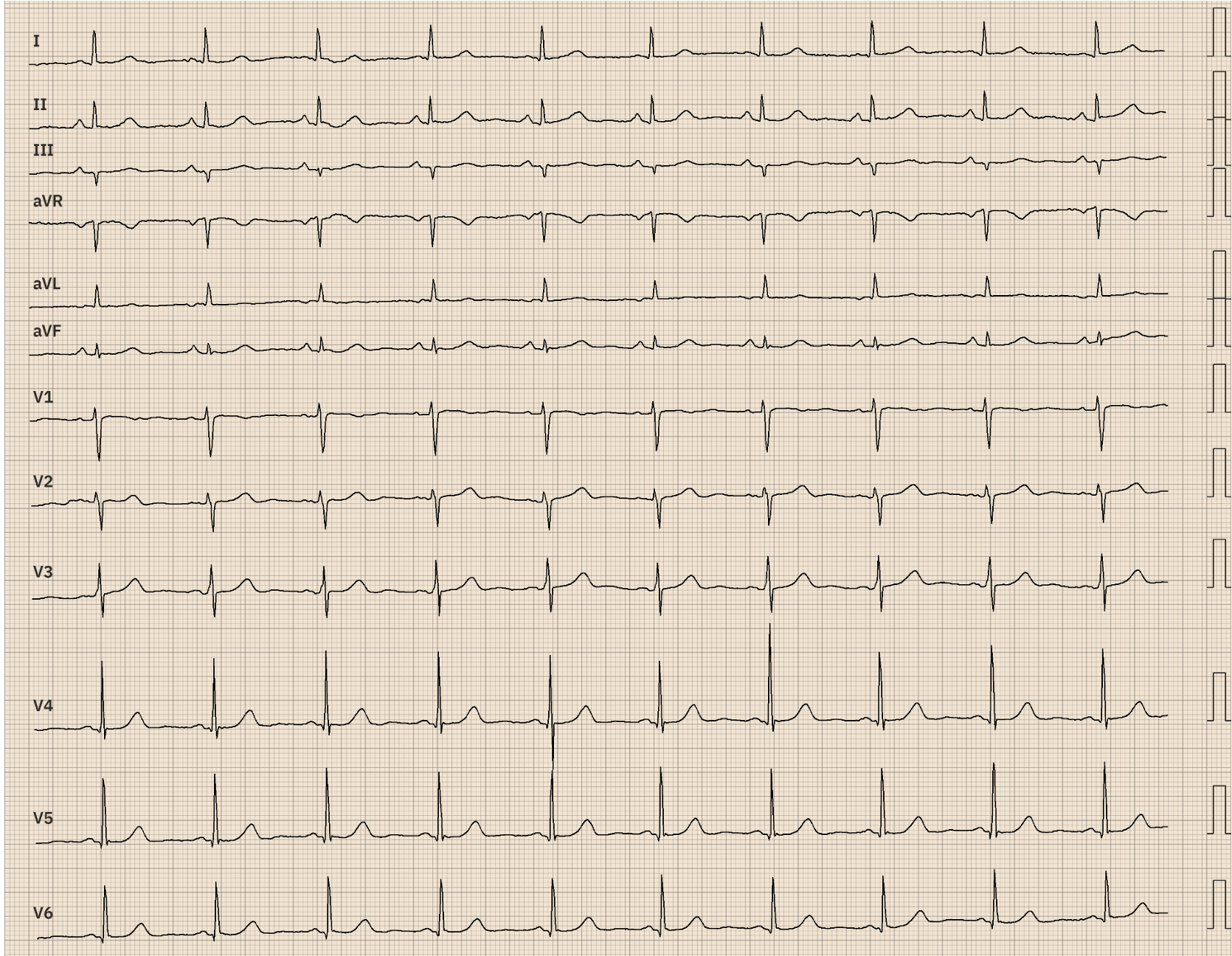

Supplement: ytaf006_Supplementary_Data [file ytaf006_supplementary_data.zip › Figure S1.jpg]
